# Genito-Urinary and Syphilitic Diseases

**Published:** 1897-08

**Authors:** Byron H. Daggett

**Affiliations:** Buffalo, N. Y.


### GENITO-URINARY AND SYPHILITIC DISEASES.

CONDUCTED BY BYRON H. DAGGETT, M. D., Buffalo, N. Y.

### THE CLINICAL FEATURES OF CHRONIC INTERSTITIAL NEPHRITIS AND CHRONIC PARENCHYMATOUS NEPHRITIS, DIFFERENTIALLY CONSIDERED.

PURDY (*The Medical Record*) says chronic interstitial nephritis, sometimes called renal cirrhoasis or granular atrophy of the kidney, pursues a chronic course from the beginning. At the beginning there are no active or pronounced symptoms. It occurs under two forms, hereditary and acquired. The hereditary form may appear in subjects of any physical type and at variable age, while the acquired type usually appears at middle or advanced ages in robust subjects, who live inactive lives and who gratify inordinate appetites upon animal food. Its earlier symptoms are transient, recurrent headaches and a varying degree of vertigo. The pulse is full, hard and incompressible, and often slower than normal. The normal cardiac sound is accentuated and the sphygmograph shows tense radial pulse. The cardiac apex approaches the left nipple line and, as the disease advances, may extend beyond it. Obstinate epistaxis and frequent urination are frequently, almost invariably, present. The quantity of urine is progressively increased at night and diminished by day, until the later stages, when this condition is reversed. There are usually traces of albumin present, but not always. Cases have been reported to pass through uremic convulsions without the presence of albumin and survive for several years.

Dr. Purdy gathered and reported 191 cases of Bright's disease, some of them having been treated in hospitals in Great Britain and in this country, in which the diagnosis was only made after the post mortem (*Medical Journal and Examiner*, May, 1885). Fifteen to 20 per cent. of the cases of chronic interstitial nephritis do not show albumin in the urine at times and often for long periods of time, regardless of the stage of the disease. Such cases baffle diagnosis and confound life insurance associations.

The urine, however, from the beginning contains casts that are distinctive in character, being narrow, slender and long, showing that they are found in the smaller uriniferous tubes beyond Henley's loops, beneath the capsules of the kidney, the seat of the primary anatomical changes of this disease. These casts are few in number, hyaline or transparent, and require careful shading and delicate focusing to bring them into view. There is little urinary sediment, the chlorides are normal in quantity and the phosphates reduced. The urea may be reduced slightly or it may remain normal in quantity.

After the disease has progressed for a number of years the symptoms become more pronounced. Headache and vertigo are more severe and frequent ; dyspnea and palpitation of the heart appear ; the skin becomes cyanotic and the arteries more prominent and tortuous ; atheroma and increased pulse tension are manifested ; appetite and digestion are impaired, associated with diarrhea and increasing dyspnea. Urination at night is more frequent and apoplectic symptoms are not uncommon. Changes in the retina now appear, more or less impairing vision, and dropsy remains absent.

If the cardio-vascular changes are prominent, albumin is plainly present. The small hyaline casts increase in number and the small-sized dark granular casts now begin to appear ; the large broad ones are not found. The urinary sediment is small in quantity.

In the late stages of chronic interstitial nephritis there are two distinct types. One is characterised by marked cardio-vascular manifestations, which obscure and overshadow the causative renal disease ; in the other these changes are slight or totally absent. The cardiac may outrun the renal lesion and terminate life years before the kidney lesion would otherwise cause death.

As the disease advances, the symptoms become more aggravated, viz. : cyanosis, dyspnea, atheroma, dropsy, apnea, cough and the like. The pulse becomes weak, compressible and even dicratic.

The tongue becomes coated with brownish fur and the breath laden with uric odor. The urine decreases in quantity and albumin becomes a persistent and increasing factor ; finally, drowsiness, convulsions, Cheyne-Stokes respiration, coma and death ends the scene.

In the second form of chronic interstitial nephritis, the cardiovascular changes are slight or absent, while there is progressive deterioration of the general health, strength and mental faculties, with headaches, forebodings and childish notions. There is general bodily and mental deterioration, without the manifestations of pronounced active disease. There is no cough, dyspnea or dropsy, but rheumatic and gouty symptoms are usually present, vision fails and the oculist is often the first to diagnosticate the disease. The urine is quite normal in quantity and the few hyaline casts are found only after diligent search. These are the latent cases, lasting often ten to fifteen years and diagnosticated as " renal insufficiency."

Chronic parenchymatous, tubal or diffuse nephritis is essentially different in all its more prominent clinical features. Invalidism is marked from the beginning. Anemia, dropsy, pallor and puffiness of the features and the extremities are early symptoms, calling attention to the seat of the disease. Debility, weakness, impaired appetite and dropsy appear early. Dropsy begins usually in the feet, extends through the cellular connective tissue and involves one or more of the great serous cavities. Visual disorders are comparatively rare. The urine is cloudy and deposits a large amount of sediment. Albumin is always present, frequently in large bulk. The urinary sediment is made up of epithelium, pus, blood, the products of cellular degeneration and numerous casts. The casts are of all varieties, but most of them are large or nearly straight, indicating that they were formed in the middle zone of the cortex or in the straight tubes of the pyramids of Malpighi. In the very chronic cases fatty casts are found. In typical and unmixed cases the differential diagnosis of these different forms of nephritis is easily made.

The differential clinical features of these two diseases :

| | |
|---|---|
| Chronic interstitial nephritis occurs in robust health and about middle life. | Chronic parenchymatous nephritis occurs in children and young adults impaired in health. |
| Patient apparently healthy in the early course of this malady. | Health impaired from the beginning, with debility, anemia and dropsy as prominent features. |

| | |
|---|---|
| Cardio-vascular changes begin early. | These changes are limited to the later stages of the disease. |
| Dropsy, if present, only appears in the later stages and is limited to the lower extremities and abdomen. | Dropsy is an early and prominent manifestation and continues throughout. |
| Cyanosis, dyspnea, cough, vertigo, increased pulse tension and nocturnal micturition are common. Uremia and visual disorders are much more common. | Absent or limited to the last stages. |
| Albumin always small in quantity, a mere trace and sometimes absent. | Always present and in large amounts. |
| Urinary sediment is slight in quantity, consisting of hyaline casts and some morphological products. | Sediment abundant, rich in casts and morphological products. The casts are large and present the features of active degenerative changes. |

---

### CONTRAINDICATIONS TO THE EMPLOYMENT OF SODIUM SALICYLATE IN RHEUMATISM.

M. JACEOUND (*Journal de Medecine et de Chirurgie Pratiques*, February 10, 1897,) states that salicylate of sodium in acute articular rheumatism does not cure the visceral localisation of this malady; that it does not palliate the manifestations of visceral involvement, but that it may even favor their production. This drug should be suspended if delirium is present, whether due to the malady or arising from any other cause.

Especially should this remedy be discontinued in case of cardiopulmonary localisation, as it has a very depressing action on the heart. The salicylate acts on the pains and on the fever, but has no modifying action upon the visceral localisations. This remedy will hasten, if not promote myocarditis.

Statistics show that these lesions are not cured or even prevented by the salicylate treatment.—*N. Y. Med. Journal*, April 3, 1897.

---

### TREATMENT OF GONORRHEA WITH SILVER SALTS.

KREISSEL, of Chicago, (*Chicago Medical Reporter*, February, 1897,) says: Several years ago Schaffer (Breslau) conducted a series of experiments for the purpose of ascertaining in what way and to what degree the usual antigonorrheal remedies affected the gonococci in regard to checking their development and impairing the culture medium.

No germs developed on the surface of the serum-agar after treatment with a 1 to 2,000 solution of nitrate of silver. The germs developed abundantly on the argentamine-serum-agar 1 part to 1000. The argentamine forms insoluble compounds with organic substances. Argonin, 15 to 20,000 solution, caused suspension of development (Meyer) in fifteen minutes. Sulpho-carbolate of zinc and alumnol were ineffective. Four per cent. solution of ichthyol after ten minutes' contact with the culture medium was inefficient.

Argentamine and nitrate of silver are powerful germicides, but are irritants to the living tissues. Irritation, by increasing inflammation, promotes the development of gonococci. Argonin, being non-irritating, is the very remedy for the early stages of acute gonorrhea. The earlier it is used the better the result. Argonin not only does not cause irritation it relieves chordee and smarting within forty-eight hours and changes the secretion to a scanty sero-mucous discharge within five days. Five to 7 per cent. injections should be used once each day for from twelve to eighteen days. The catarrh and hypersecretion following the disappearance of the gonococci should be treated with astringents, irrigation and the like.

Tests of cure—namely, either venery, the use of liberal amounts of beer, the passage of the olive-tipped bougie or deep injections of silver nitrate were applied in selected cases.

---

### SECTION OF THE VAS DEFERENS.

NAVÉ JASSERAND (*Lyon Medical Journal*) gives the results carefully observed in forty-six vasectomies. He alleges that section of the vas rarely affects the size of the prostate, but it does relieve vesico-prostatic congestion and consequent urinary retention, spasm and pain. Reduction of the size of the prostate is not essential for the relief of these symptoms.

If the retention has not been of too long standing, urination becomes entirely normal, and in all cases pain and strangury are relieved. The operation produces sterility without destruction of the virile function. The immediate unfavorable results, retention, shock and mental disturbance are transitory and not severe. The operation is so slight that all patients bear it easily and are not confined to the bed. It does not contraindicate any subsequent surgical procedure. It is especially indicated in the middle period of prostatism, when congestion is inciting cystitis, hematuria and retention. It diminishes pain in all stages of prostatic abnormalities.—*Bulletin Med.*, November 22, 1896.

Reginald Harrison, of London, and others report favorable results following vasectomy. Herhold, in Germany, reports forty-four cases in which thirty were cured and none died. Drézigné has performed the operation in twenty-two cases with notable improvement in every case. Operation on one side, only, produced no effects.

Seventy-five cases of vasectomy are reported with a high degree of success and no danger. Carlier reports seven vasectomies and states that he believes that favorable results are due to the methodical after-treatment. Loumeau reports four vasectomies and alleges that the only result was a cessation of frequent recurrence of epididymitis in two of these cases.

The abstractor herewith adds four cases of vasectomy, three of which were performed in the service of Dr. B. H. Daggett at the Erie County Hospital, and the results fully confirm the reports made by Jasserand. All the urinary disturbances were relieved, except in one case, and he was an old and feeble man, and even in this case all the urinary symptoms resulting from senile hypertrophy were relieved, excepting that he was required to urinate three times during the night. Pain, strangury and turbid urine disappeared.

